# Household Food Insecurity Is Not Associated with BMI for Age or Weight for Height among Brazilian Children Aged 0–60 Months

**DOI:** 10.1371/journal.pone.0045747

**Published:** 2012-09-21

**Authors:** Gilberto Kac, Michael M. Schlüssel, Rafael Pérez-Escamilla, Gustavo Velásquez-Melendez, Antônio Augusto Moura da Silva

**Affiliations:** 1 Observatório de Epidemiologia Nutricional, Instituto de Nutrição Josué de Castro, Universidade Federal do Rio de Janeiro, Rio de Janeiro, Brazil; 2 Yale School of Public Health, New Haven, Connecticut, United States of America; 3 Escola de Enfermagem. Departamento de Enfermagem Materno-infantil em Saúde Pública. Universidade Federal de Minas Gerais, Belo Horizonte, Brazil; 4 Department of Public Health, Federal University of Maranhão, São Luís, Maranhão, Brazil; Instutite of Agrochemistry and Food Technology, Spain

## Abstract

We examined the association between Household Food Insecurity (HFI), weight for height z-score (WHZ) and Body Mass Index for age z-score (BMI-Z) in a representative sample of children 0–60 months of age (n = 3,433) in five Brazilian geographical regions. Data were derived from the 2006–07 Brazilian Demographic and Health Survey. HFI was measured with the Brazilian Food Insecurity Scale. Associations were estimated using multiple linear regression models (ß coefficients and 95% CI) taking into account the complex sampling design. Interaction terms between HFI and geographical region and HFI and child sex and child age were assessed. The weighted prevalence of any level of HFI was 48.6%. Severe food insecurity was more prevalent among children from the North region (16.8%), born from mothers with <4 years of schooling (15.9%) and those from families with ≥3 children (18.8%). The interaction between HFI and geographical region was non-significant for BMI-Z (P = 0.119) and WHZ (P = 0.198). Unadjusted results indicated that HFI was negatively associated with BMI-Z (moderate to severe HFI: ß = −0.19, 95% CI: −0.35 - −0.03, P = 0.047), and WHZ (moderate to severe HFI: ß = −0.26, 95% CI: −0.42 - −0.09, P = 0.009). Estimates lost significance after adjustments for key confounders such as mothers' skin color, mothers' years of schooling, place of household, household income quartiles, mothers' smoking habit, mothers' marital status, number of children 0–60 months in the household, and birth order. HFI is unrelated to weight outcomes among Brazilian children 0–60 months.

## Introduction

Women living in households with food insecurity (HFI) seem to be at increased risk of obesity [1], [2]. However, among children, support for an association between HFI and weight status has been mixed [3]–[5]. A small number of studies have found that children living in food insecure households are more likely to be overweight or obese [6]–[9], however other studies have not found an association between HFI and weight status [10], [11] or have found that HFI is associated with lower risk of pediatric obesity [12], [13]. Some studies that reported that HFI is associated with a higher risk of overweight found that this association was present only among girls but not among boys [6], [7]. Most of these studies were carried out in high-income countries and combined children with adolescents in the analysis [2], [4], [9]. In low and middle-income countries results are also mixed [14]–[19].

Our research group has previously documented associations between HFI and obesity risk among adult and adolescent women using data from the latest Brazilian Demographic and Health Survey (DHS) [1], [20]. However the relationship between HFI and the risk of childhood obesity among different sex and age-subgroups of Brazilian children remains to be elucidated. The answer to this question is essential to understand if there is a relationship between HFI and obesity risk across different stages of the life cycle.

The objective of this paper is to examine the association between HFI, weight for height z-score (WHZ) and Body Mass Index for age z-score (BMI-Z) in a representative sample of children 0–60 months of age in five Brazilian geographical regions after adjusting for key potential confounders. Because Brazil is a highly diverse country we also examined the interaction between HFI and geographical region on each of these anthropometric outcomes.

## Methods

### Ethics Statement

The project was approved by the Research Ethics Committee of the Sexually Transmitted Diseases/AIDS Reference and Training Centre of the Health Secretariat of the state of São Paulo.

### Study design and sampling

The data were derived from the third wave of the DHS, conducted in Brazil, in 2006–07. DHS was a population-based cross-sectional study with a nationally representative sample of women of reproductive age and mothers of children aged 0–60 months. DHS included both household- and individual- but no community- level variables, and has a complex sampling design. The sample units were selected in two stages. The first stage involved selecting the census sectors, which were the primary sampling units. The second stage involved selecting the survey participants within each household. Ten sampling strata were defined based on a combination of urban *vs.* rural areas and the five Brazilian geographical regions. The respondents' sampling weights were derived from the household sampling weights and took into account the probability that there may be more than one eligible woman per household. The weights were adjusted for non-response within households and were calibrated based on official population estimates released by Brazilian Institute of Geography and Statistics.

Structured questionnaires were applied to the mothers or caregivers of the children through in-person interviews and children's anthropometric measures were taken. Data collected included socioeconomic status, lifestyle variables, reproductive history, and HFI.

### Anthropometric data and study sample

Weight and length or heights of all eligible children in the selected households were measured according to the recommendations of the World Health Organization [21]. These measurements were conducted twice for each subject, and the mean value was calculated. Weight was measured using an electronic scale (Dayhome ®) with a 100 g precision, which was calibrated at the beginning and at the end of each working day. Length or height was measured using stadiometers made locally for the purpose of the study with 1 mm precision that were calibrated at the beginning and at the end of each working day. BMI was calculated as weight (kg)/length or height (m^2^). The anthropometric assessments were performed by evaluators who had completed high school, and who received a 32-hour training course on child anthropometry, focusing mainly on standardizing length and height measurements. The evaluators' performance was assessed against a gold-standard anthropometrist, and only those demonstrating adequate accuracy and precision participated on the data collection.

The initial analytical sample was formed by 5,146 children, of both sexes aged 0–60 months, living in 4,108 households. The analytical sample considered for these analyses included the 3,527 subjects that had available data for all key variables (age, sex, weight, length or height and HFI), and covariates. The variables with the most missing data were weight and height/length (n = 1,184) and family income. We excluded those individuals classified as outliers (n = 94), assuming that their anthropometric measures were physiologically implausible, using the following criteria: Height for age z-score (HAZ)<−5 and >+3, Weight for age z-score (WAZ)<−5 and >+5 and WHZ<−4 and >+5 (21). Although HAZ and WAZ were not directly measured, it was necessary to identify outliers for these indicators based on physiologically implausible limits for weight or height measures, as both are needed for estimating the study outcomes (BMI-Z and WHZ).

The final sample comprised 3,433 children aged 0–60 months. The age categorization (0–24; 25–60 months) was implemented in order to group children with similar anthropometric data collection protocols for length (<24 months) or height (≥24 months), and also due to expected differences in growth patterns among these age groups.

### Outcome and independent variables

In our analyses we included two different anthropometric outcomes, first to check results consistency between outcomes and second to fully examine the association between HFI and different measures of weight status. BMI-Z and WHZ were calculated according to the World Health Organization (WHO) growth reference standards [22]. BMI-Z can measure either excess or insufficient weight for a certain length or height. WHZ is an indicator of body fat with higher values representing excess weight or obesity. All indicators were expressed as sex and age (in months) specific z-scores, and were analysed as continuous variables.

HFI level (security, mild insecurity, moderate insecurity, severe insecurity) was the key independent variable and was measured with the previously used and extensively validated Brazilian Food Insecurity Scale (EBIA). EBIA represents an adaptation of the US Household Food Security Survey Module (US-HFSSM), developed during the early 90's and first fielded in the 1995 US Current Population Survey. The detailed description of the adaptation and validation of the EBIA scale can be found elsewhere [23], [24], but it is important to state that several validity criteria (content, face, predictive and convergent) were met. The EBIA is composed of 15 dichotomous (yes/no) questions that evaluate food insecurity experiences, ranging from the worry or concern that the household may run out of food, to sacrificing the quality of the diet and to restricting the amount of food consumed, and ultimately going for a whole day with little or no food due to economic limitations. Each household is assigned a summative food insecurity score based on the number of affirmative responses to the scale items. Households were classified either as food secure (HFI score = 0), mildly food insecure (score = 1–5), moderately food insecure (score = 6–10), or severely food insecure (score = 11–15).

As in our previously published studies with adolescent [20] and adult [1] women, the other key confounders included in the analyses were: mother's self-reported skin colour/ethnicity (white, black, brown, yellow, indigenous); maternal schooling in years (0–4, 5–8, ≥9); place of household (urban, rural); geographical region of the household (North, Northeast, Southeast, South, Midwest); quartiles of family income (in Brazilian *reais*, about US$ 0,47 per *real* in 2006–07); mothers' smoking habit (yes, no), mother's marital status (single/widowed/divorced, married or cohabiting), number of children 0–60 months in the household and birth order. These covariates were selected based on theoretical and empirical considerations and allows for direct comparison between the findings of the present study with those of previous studies [1], [20].

### Post-hoc sample size calculations

Since the number of children available for analysis was fixed and based on the number available on the DHS database post-hoc sample size calculation was performed. A sample size of 157 children in each HFI group is able to detect a difference of 0.1 z-score between two HFI groups for each anthropometric indicator, working with 80% of power and assuming a standard deviation of 0.4 z-score based on our sample data. Sample size calculations were performed in Stata 12.0 using the *sampsi* command.

### Analytical sample bias analyses

Analytical sample bias analyses were conducted using an weighted (which took into account the complex sample design) chi-square test for categorical variables in order to understand if the overall profile of the anthropometric outliers (n = 94) and those excluded due to missing key variables (n = 1,613), differed from those included in the analyses (n = 3,433). Comparisons were performed in regards to HFI level prevalence, geographical region and other key confounders.

### Statistical Analyses

We first analysed HFI (mild, moderate and severe food insecurity) prevalence distribution according to key confounding variables. Comparisons were performed using weighted chi-square test for differences in proportions. In a second stage, we examined the mean values (with 95% CI) for the two outcomes (WHZ and BMI-Z), according to HFI. Third, interaction terms between HFI and geographical region, child gender and age on each of the two anthropometric outcomes were tested.

We conducted crude and adjusted linear regression analyses (ß coefficients and 95% CI), adjusting for key confounders and having WHZ and BMI-Z as outcomes. Separate independent crude and adjusted linear regression models were estimated. Estimates were weighted and the 95% CI corrected to take into account the complex sampling design by means of *svy* commands in Stata (Stata 12.0 software, StataCorp, Texas, USA). Moderate and severe HFI categories were combined to increase the precision and study power.

Lastly, we performed three further analyses to assure that the analytical approach used has produced unbiased and valid results. First we re-analyzed the data including only one child per household, the youngest child (n = 2849, representing 83% of the original sample), considering that 18% of the households had more than one child. Further we run a model with adjustment for mother's weight and height and finally, since mother's schooling or income could act alongside HFI or via HFI on BMI-Z or WHZ we have run models removing these variables.

## Results

The likelihood of being excluded as outliers according to the anthropometric z-score values (n = 94) or due to missing information for the key variables (n = 1,619) was higher for children living in food secure households, from the South geographical region, for those living in rural areas and for children of mothers' with schooling <4 years, of white skin color and single or divorced, when compared to those included (n = 3,433) (**Table S1**).

The weighted overall prevalence of any level of HFI was 48.6% (results not shown in tables). Severe HFI was more prevalent among children living in the North region, living in lower income families, with black or brown skin mothers, with mothers who had 0–4 years of schooling, and living in households with ≥3 children of age (**Table 1**).

**Table 1:** Frequency distribution of Household Food Insecurity (HFI) levels according to socio-demographic variables.

	Food security		HFI level						
			Mild		Moderate		Severe		*P* 2
	n	%	n	%	n	%	n	%	
Geographical region of household									<0.001
North	312	39.43	193	24.15	153	19.63	154	16.79	
Northeast	250	42.17	203	28.55	155	21.07	73	8.21	
Southeast	389	55.19	196	33.36	60	9.45	19	2.00	
South	385	67.20	154	23.48	43	5.88	20	3.44	
Midwest	374	59.31	198	27.08	70	9.92	32	3.69	
Place of household									0.277
Urban	1,163	51.16	653	30.41	319	12.89	184	5.54	
Rural	547	52.65	291	24.66	162	16.29	114	6.40	
Family income (quartiles)									<0.001
1	337	30.99	334	32.66	275	24.67	218	11.68	
2	479	44.20	321	34.99	150	14.84	60	5.97	
3	453	64.60	210	29.71	46	4.80	15	0.89	
4	441	85.15	79	12.79	10	1.70	5	0.36	
Mother's skin color									<0.001
White	709	61.58	274	24.96	103	8.92	62	4.54	
Black	133	38.75	109	37.23	65	19.42	28	4.60	
Brown	796	47.89	517	30.52	187	15.11	179	6.48	
Yellow	51	55.60	24	28.14	11	11.81	4	4.45	
Indigenous	21	23.16	20	34.01	15	22.53	25	20.30	
Mother's years of schooling									<0.001
≥9	904	66.91	329	24.77	83	5.98	40	2.34	
5–8	530	42.87	367	33.77	240	18.84	93	4.53	
0–4	276	30.98	248	31.92	158	21.17	165	15.92	
Mother's smoking habit									0.001
No	1,527	54.44	796	27.53	383	12.62	238	5.41	
Yes	183	35.13	148	39.33	98	18.24	60	7.30	
Mother's marital status									0.194
Single/window/divorced	202	42.11	109	34.26	72	19.07	45	4.56	
Married or cohabiting	1,508	52.72	835	28.70	409	12.73	253	5.85	
Sex									0.820
Boys	874	50.42	476	29.89	254	14.19	146	5.50	
Girls	836	52.53	468	28.81	227	12.74	152	5.92	
Age (months)									0.568
0–24	1,018	52.71	550	29.01	255	13.12	150	5.16	
25–60	692	49.61	394	29.89	226	14.03	148	6.47	
Birth order									<0.001
1	777	58.06	392	29.27	124	9.88	49	2.79	
2	556	52.40	260	28.27	128	14.79	62	4.54	
≥3	377	36.22	292	31.01	229	19.48	187	13.29	
Number of children 0–60 months living in the household									<0.001
1	1,131	56.93	558	28.52	202	10.51	107	4.04	
2	479	42.24	291	32.55	181	17.96	115	7.25	
≥3	100	28.60	95	23.94	98	28.63	76	18.83	

Demographic Health Survey, Brazil 2006.^1^

1 The column “n” presents real sample values and the column “%” presents values expanded to the Brazilian population.

2 P values refer to the Chi-square test for differences in proportions.

BMI-Z mean values were lower for children who lived in moderate to severe food insecurity compared with mild and food-secure households. A similar pattern was observed for WHZ (**Table 2**).

**Table 2:** Body mass index for age (BMI-Z) and weight for age (WHZ) means according to the Household Food Insecurity (HFI) level.

	BMI-Z			WHZ		
	*n*	Mean	95% CI	*n*	Mean	95% CI
HFI level						
Food security	1,710	0.52	0.44–0.61	1,170	0.48	0.40–0.57
Mild food insecurity	944	0.50	0.39–0.61	944	0.45	0.34–0.56
Moderate+severe food insecurity	779	0.33	0.20–0.46	779	0.22	0.08–0.37
Total	3,433	0.48	0.42–0.54	3,433	0.42	0.36–0.49

Demographic Health Survey, Brazil 2006.^1^

1 Estimates were weighted and 95% CI were corrected to take into account the complex sampling design.

**CI:** confidence interval.

We did not find a significant interaction between HFI and geographical region for BMI-Z (P = 0.119) or WHZ (P = 0.198). Thus, all analyses were conducted for the country as a whole and were not stratified by geographical region (**results not shown in tables**).

HFI was negatively associated with BMI-Z (moderate to severe HFI: ß = −0.19, 95% CI: −0.35 - −0.03, P = 0.047), and WHZ (moderate to severe HFI: ß = −0.43, 95% CI: −0.26 - −0.09, P = 0.009) in the unadjusted analysis. Estimates lost significance after analysis were adjusted for mothers' skin color, mothers' years of schooling, place of household, household income quartiles, mothers' smoking habit, mothers' marital status, number of children 0–60 months in the household, and birth order (**Table 3**).

**Table 3:** Crude and adjusted linear regression models for the effect of the Household Food Insecurity (HFI) level on body mass index for age (BMI-Z) and weight for height (WHZ).

		BMI-Z					
	*n*	crude ß	95% CI	*p* 2	adjusted^3^ ß	95% CI	*p* 2
Household Food Insecurity	3,433			0.047			0.501
Security	1,710	reference	-		reference	-	
Mild insecurity	944	−0.02	−0.16–0.11		0.01	−0.12–0.14	
Moderate+severe insecurity	779	−0.19	−0.35–−0.03		−0.09	−0.27–0.08	

		WHZ					
	*n*	crude ß	95% CI	*p* 2	adjusted^3^ ß	95% CI	*p* 2
Household Food Insecurity	3,433			0.009			0.292
Security	1,710	reference	-		reference	-	
Mild insecurity	944	−0.03	−0.17–0.11		0.02	−0.11–0.15	
Moderate+severe insecurity	779	−0.26	−0.42–−0.09		−0.13	−0.32–0.05	

Demographic Health Survey, Brazil 2006.^1^

1 Estimates were weighted and 95% CI were corrected to take into account the complex sampling design;

2 p values refers to the Wald test for multiple comparisons;

3 Adjusted for geographical region of household, place of household, household income quartiles, mothers' skin color, mothers' years of schooling, mothers' smoking habit, mothers' marital status, child sex, age, order of birth and number of children 0–60 months of age living in the household.

**CI:** confidence interval.

Further analyses including only one child per household revealed that key results, specifically the unadjusted and adjusted coefficients for BMI-Z and WHZ barely changed and most importantly, kept the direction of the association. The model adjusted for mother's weight and height did not affect the main results. The same happened when mother's schooling and income were removed (data not shown, available on request).

## Discussion

The main finding of this study is an unadjusted relationship between HFI and children's BMI-Z for age or WHZ. However, this association lost significance after adjusting for known confounders including mother's skin color, mother's years of schooling, place of household, quartiles of family income, mother's smoking habit, mother's marital status, number of children 0–60 months in the household and birth order. It is important to note however that overweight and obesity prevalence's in Brazil are still low among children 0–60 months. These findings are in partial agreement with a previous analysis [16] and add to it substantially by carefully examining the relationships within Brazilian geographical regions, by including BMI-Z and WHZ as outcomes of interest, and by adjusting for the same confounders included in a previously published analyses examining the HFI-obesity relationship among adolescent [20] and adult [1] Brazilian women. However, the current findings are not in full agreement with studies conducted in other low- and middle-income countries such as Colombia [14], [25]–[26], Pakistan [27], Jamaica [17], Korea [18] and Mexico [19]. This great heterogeneity in results may be explained by the inclusion of different child age groups across studies, the use of different scales to measure HFI and different approaches to analyze food insecurity data (e.g., continuous vs. categorical), and/or the possibility that the relationships between HFI and child anthropometric indicators is highly context specific. Longitudinal designs are needed to disentangle these complex relationships. These studies will need to take into account potential effect modification by geographical region, gender, age and the child birth-weight [15], [17].

Following a life-course approach, we have previously documented in Brazil, that HFI is associated with obesity risk among adult and with excessive weight among adolescent women investigated by the 2006 DHS [1], [20]. Thus, an important question becomes: why the association between HFI and higher BMI-Z or WHZ mean values was not found among any of the sub-groups of Brazilian children 0–60 months of age that we examined?

A potential explanation for the lack of association between HFI and higher BMI-Z means values in Brazilian children studied by the DHS relies on the different velocities and stages of the nutrition transition experimented by diverse socio-economic status sub-groups and individuals at different periods of the life-cycle, during the last decades. It has already been documented earlier that the dynamics in regards to the occurrence of a shift from under-nutrition to obesity tends to follow a pattern that usually occurs first in adults, followed by adolescents, and only later in time by children [28]. This nutrition transition pattern has been extensively documented for the Brazilian population having as reference some of the most important nationally representative surveys from the last 20 years [28], [29].

On the other hand, the deleterious effect of the HFI represented mainly by an increased prevalence of obesity or higher BMI-Z mean population values, may constitute a feature of a very advanced stage of the nutrition transition, and thus has been observed only in adults and adolescents, the first groups to achieve the new pattern, but not in children 0–60 months, the group that usually suffers the nutrition transition effects at a later stage. We can conclude that the nutrition transition happening in Brazil may still be having less of an impact among young children when compared to older children, youth and adults living in food insecure households, when the country as a whole is considered. A cross-sectional study conducted in Pelotas, southern of Brazil, thus a more developed region, has shown that excessive weight for children under 5 years, in families living in food insecurity [30]. These results support our hypothesis. Alternatively the high nutrient demands that young children have per unit of body weight may also partially explain why the obesity risk/higher BMI-Z mean values, among children as a result of exposure to HFI is differentially affected in relationship to adult women. A recent study conducted on a vulnerable urban area of Rio de Janeiro, Brazil, has shown that children between 6 and 30 months living in food insecure households were more likely to have lower quality diets, characterized by highly energy dense foods and low protein content [31]. It is possible that the relatively high caloric requirements per unit of body weight that children of this age have, may “protect” them against becoming obese, even if they are exposed to cheap energy dense diets that may result from HFI. It is important to monitor if and how HFI coping mechanisms differ (e.g. dietary adaptation) across age groups in the context of the nutrition transition.

The cross-sectional nature of the study, as well as the lack of information on coping mechanisms associated with diverse levels of HFI should be considered as a limitation. The evidence for a relationship between food insecurity and nutritional status is still inconsistent, as almost all studies are cross-sectional, precluding the possibility of establishing the temporality of events. In this regard, it is essential to conduct prospective studies that collect data on HFI status across time, as well as information as to how children and adults within the household cope with HFI changes. Special attention needs to be paid to dietary intake and physical activity adaptations. Ultimately the goal is to better understand how these coping behaviours influence body fat accumulation at different stages of the life cycle and across countries undergoing different phases of the nutrition transition. In addition it is important to acknowledge that complex surveys as DHS often times have substantial missing data in key self-reported confounders such as birth weight and digit preference biases in indicators such as breastfeeding duration. Our study is prone to some sort of selection bias, because there were statistically significant differences when comparing included vs. excluded subjects due to missing values. The likelihood of being excluded was higher for children with food security, from the South geographical region, for children of mothers' with schooling <4 years and single or divorced. Thus, the results need to be interpreted with caution.

This study detected that there was no association between HFI and BMI-Z or WHZ values in Brazilian children 0–60 months of age. These results together with empirical evidence from Brazil and other countries suggests that the nutrition transition phase at which countries are needs to be taken into account when interpreting findings from studies examining the relationship between HFI and obesity risk across the life cycle.

## Supporting Information

Table S1 **Frequency distribution of main investigated variables between those who were included in the study and those who were not.** Demographic Health Survey, Brazil 2006.
(DOC)

## References

[1] Velasquez-Melendez G, Schlussel MM, Brito AS, da Silva AA, Lopes-Filho JD, et al (2011) Mild but not light or severe food insecurity is associated with obesity among Brazilian women. J Nutr 141: 898–902. 21389183 10.3945/jn.110.135046

[2] Institute of Medicine (2011) Hunger and Obesity: Understanding a Food Insecurity Paradigm: Workshop Summary. Washington, DC: The National Academies Press. 24983070

[3] Larson NI, Story MT (2011) Food insecurity and weight status among U.S. children and families: a review of the literature. Am J Prev Med 40: 166–173. 21238865 10.1016/j.amepre.2010.10.028

[4] Eisenmann JC, Gundersen C, Lohman BJ, Garasky S, Stewart SD (2011) Is food insecurity related to overweight and obesity in children and adolescents? A summary of studies, 1995–2009. Obes Rev 12: e73–83 DOI: 10.1111/j.1467-789X.2010.00820.x. 21382151 10.1111/j.1467-789X.2010.00820.x

[5] Pérez-Escamilla R (2012) Food insecurity and hunger in children: Impact on physical and psycho-emotional development. In: Ross C, Caballero B, Cousins R, Tucker K, Ziegler T, editors. Modern Nutrition in Health and Disease. 11th ed. Baltimore, MD: Lippincott Williams & Wilkins (In press).

[6] Metallinos-Katsaras E, Sherry B, Kallio J (2009) Food insecurity is associated with overweight in children younger than 5 years of age. J Am Diet Assoc 109: 1790–1794. 19782181 10.1016/j.jada.2009.07.007

[7] Jyoti DF, Frongillo EA, Jones SJ (2005) Food insecurity affects school children's academic performance, weight gain, and social skills. J Nutr 135: 2831–2839. 16317128 10.1093/jn/135.12.2831

[8] Alaimo K, Olson CM, Frongillo EA Jr (2001) Low family income and food insufficiency in relation to overweight in US children: is there a paradox? Arch Pediatr Adolesc Med 155: 1161–1167. 11576013 10.1001/archpedi.155.10.1161

[9] Casey PH, Simpson PM, Gossett JM, Bogle ML, Champagne CM, et al (2006) The association of child and household food insecurity with childhood overweight status. Pediatrics 118: e1406–1413 DOI: 118/5/e1406 [pii] 10.1542/peds.2006-0097. 17079542 10.1542/peds.2006-0097

[10] Gundersen C, Garasky S, Lohman BJ (2009) Food insecurity is not associated with childhood obesity as assessed using multiple measures of obesity. J Nutr 139: 1173–1178. 19403713 10.3945/jn.109.105361

[11] Bhargava A, Jolliffe D, Howard LL (2008) Socio-economic, behavioural and environmental factors predicted body weights and household food insecurity scores in the Early Childhood Longitudinal Study-Kindergarten. Br J Nutr 100: 438–444. 18275621 10.1017/S0007114508894366

[12] Rose D, Bodor JN (2006) Household food insecurity and overweight status in young school children: results from the Early Childhood Longitudinal Study. Pediatrics 117: 464–473. 16452367 10.1542/peds.2005-0582

[13] Jones SJ, Jahns L, Laraia BA, Haughton B (2003) Lower risk of overweight in school-aged food insecure girls who participate in food assistance: results from the panel study of income dynamics child development supplement. Arch Pediatr Adolesc Med 157: 780–784. 12912784 10.1001/archpedi.157.8.780

[14] Isanaka S, Mora-Plazas M, Lopez-Arana S, Baylin A, Villamor E (2007) Food insecurity is highly prevalent and predicts underweight but not overweight in adults and school children from Bogota, Colombia. J Nutr 137: 2747–2755. 18029494 10.1093/jn/137.12.2747

[15] Oliveira JS, Lira PIC, Maia SR, Sequeira AS, Amorim RCA, et al (2010) Food insecurity and the nutritional status of children in Gameleira, in the Forest Zone of the Brazilian Northeast. Rev Bras Saude Mater Infant 10: 237–245.

[16] Reis M (2012) Food insecurity and the relationship between household income and children's health and nutrition in Brazil. Health Econ 21: 405–427. 21344538 10.1002/hec.1722

[17] Dubois L, Francis D, Burnier D, Tatone-Tokuda F, Girard M, et al (2011) Household food insecurity and childhood overweight in Jamaica and Quebec: a gender-based analysis. BMC Public Health 11: 199 DOI: 10.1186/1471-2458-11-199. 21453491 10.1186/1471-2458-11-199 PMC3078098

[18] Oh SY, Hong MJ (2003) Food insecurity is associated with dietary intake and body size of Korean children from low-income families in urban areas. Eur J Clin Nutr 57: 1598–1604. 14647225 10.1038/sj.ejcn.1601877

[19] Ortiz-Hernandez L, Acosta-Gutierrez MN, Nunez-Perez AE, Peralta-Fonseca N, Ruiz-Gomez Y (2007) Food insecurity and obesity are positively associated in Mexico City schoolchildren. Rev Invest Clin 59: 32–41. 17569298

[20] Kac G, Velasquez-Melendez G, Schlussel MM, Segall-Correa AM, da Silva AA, et al (2012) Severe food insecurity is associated with obesity among Brazilian adolescent females. Public Health Nutr 1–7 [jan 12 Epub ahead of print]. 10.1017/S1368980011003582 22251603

[21] World Health Organization (1995) Physical status: the use and interpretation of anthropometry. Geneva: World Health Organization. 8594834

[22] WHO Multicentre Growth Reference Study Group (2006) WHO Child Growth Standards: Length/height-for-age, weight-for-age, weight-for-length, weight-for-height and body mass index-for-age: Methods and development. Geneva: World Health Organization.

[23] Pérez-Escamilla R, Segall-Correa AM (2008) Food insecurity measurement and indicators. Rev Nutr 21: 15s–26s.

[24] Perez-Escamilla R, Segall-Correa AM, Kurdian ML, Sampaio Md, Marin-Leon L, et al (2004) An adapted version of the U.S. Department of Agriculture Food Insecurity module is a valid tool for assessing household food insecurity in Campinas, Brazil. J Nutr 134: 1923–1928. 15284377 10.1093/jn/134.8.1923

[25] Hackett M, Melgar-Quinonez H, Alvarez MC (2009) Household food insecurity associated with stunting and underweight among preschool children in Antioquia, Colombia. Rev Panam Salud Publica 25: 506–510. 19695145 10.1590/s1020-49892009000600006

[26] Alvarado BE, Zunzunegui MV, Delisle H (2005) Validation of food security and social support scales in an Afro-Colombian community: application on a prevalence study of nutritional status in children aged 6 to 18 months. Cad Saude Publica 21: 724–736. 15868030 10.1590/s0102-311x2005000300006

[27] Baig-Ansari N, Rahbar MH, Bhutta ZA, Badruddin SH (2006) Child's gender and household food insecurity are associated with stunting among young Pakistani children residing in urban squatter settlements. Food Nutr Bull 27: 114–127. 16786978 10.1177/156482650602700203

[28] Monteiro CA, Conde WL, Popkin BM (2002) Is obesity replacing or adding to undernutrition? Evidence from different social classes in Brazil. Public Health Nutr 5: 105–112. 12027272 10.1079/PHN2001281

[29] Kac G, Velasquez-Melendez G (2003) The nutritional transition and the epidemiology of obesity in Latin America. Cad Saude Publica 19: Suppl 1: S4–S5. 12886430

[30] Santos JV, Gigante DP, Domingues MR (2010) Prevalence of food insecurity in Pelotas, Rio Grande do Sul State, Brazil, and associated nutritional status. Cad Saude Publica 26: 41–49. 20209208 10.1590/s0102-311x2010000100005

[31] Antunes MM, Sichieri R, Salles-Costa R (2010) Food intake among children under three years of age in an area with high food insecurity. Cad Saude Publica 26: 1642–1650. 21229222 10.1590/s0102-311x2010000800017

