# Supplementary material for: Household Food Insecurity Is Not Associated with BMI for Age or Weight for Height among Brazilian Children Aged 0–60 Months
Source: PLoS One. 2012 Sep 21;7(9):e45747. doi: 10.1371/journal.pone.0045747 (PMC3448695; doi:10.1371/journal.pone.0045747)
Supplement: Table S1 — Frequency distribution of main investigated variables between those who were included in the study and those who were not. Demographic Health Survey, Brazil 2006. (DOC) [file pone.0045747.s001.doc]

| **Table S1.** Frequency distribution of main investigated variables between those who were included in the study and those who were not. Demographic Health Survey, Brazil 2006. | | | | | |
| --- | --- | --- | --- | --- | --- |
|  | Evaluated | | Excluded by missing information or being outliers of anthropometric characteristics | | |
|  |  |  |  |  |  |
|  | *n* | % | *n* | % | *p* |
|  |  |  |  |  |  |
| House-hold food insecurity |  |  |  |  | **0.022** |
| Security | 1,710 | 51.42 | 775 | 57.30 |  |
| Mild insecurity | 944 | 29.38 | 389 | 21.71 |  |
| Moderate insecurity | 481 | 13.50 | 225 | 14.23 |  |
| Severe insecurity | 298 | 5.70 | 158 | 6.76 |  |
|  |  |  |  |  |  |
| Macro-region of household |  |  |  |  | **0.011** |
| North | 812 | 11.16 | 306 | 9.08 |  |
| Northeast | 681 | 28.33 | 321 | 28.19 |  |
| Southeast | 664 | 41.70 | 319 | 37.58 |  |
| South | 602 | 11.49 | 386 | 16.39 |  |
| Midwest | 674 | 7.32 | 381 | 8.76 |  |
|  |  |  |  |  |  |
| Place of household |  |  |  |  | **0.013** |
| Urban | 2,319 | 81.98 | 1,058 | 77.39 |  |
| Rural | 1,114 | 18.02 | 655 | 22.61 |  |
|  |  |  |  |  |  |
| Family income (quartiles) |  |  |  |  | 0.132 |
| 1 | 1,164 | 32.58 | 267 | 33.35 |  |
| 2 | 1,010 | 27.17 | 214 | 26.80 |  |
| 3 | 724 | 24.08 | 135 | 18.25 |  |
| 4 | 535 | 16.16 | 129 | 21.60 |  |
|  |  |  |  |  |  |
| Mother’s skin color |  |  |  |  | **0.034** |
| White | 1,148 | 32.86 | 597 | 37.56 |  |
| Black | 335 | 11.05 | 177 | 10.09 |  |
| Brown | 1,779 | 51.16 | 783 | 45.58 |  |
| Yellow | 90 | 3.91 | 50 | 3.84 |  |
| Indigenous | 81 | 1.02 | 45 | 2.93 |  |
|  |  |  |  |  |  |
| Mother’s years of schooling |  |  |  |  | **0.013** |
| ≥ 9 | 1,356 | 44.95 | 539 | 38.78 |  |
| 5-8 | 1,230 | 36.16 | 613 | 36.50 |  |
| 0-4 | 847 | 18.89 | 525 | 24.72 |  |
|  |  |  |  |  |  |
| Mother’s smoking habit |  |  |  |  | 0.980 |
| No | 2,944 | 84.39 | 1,399 | 84.45 |  |
| Yes | 489 | 15.61 | 314 | 15.55 |  |
|  |  |  |  |  |  |
| Mother’s marital status |  |  |  |  | **< 0.001** |
| Single/window/divorced | 428 | 12.17 | 385 | 20.46 |  |
| Married or cohabiting | 3,005 | 87.83 | 1,328 | 79.54 |  |
|  |  |  |  |  |  |
| Sex |  |  |  |  | 0.767 |
| Boys | 1,750 | 52.21 | 940 | 53.06 |  |
| Girls | 1,683 | 47.79 | 773 | 46.94 |  |
|  |  |  |  |  |  |
| Age (years) |  |  |  |  | 0.730 |
| 0-2 | 1,973 | 58.68 | 1,013 | 59.52 |  |
| 3-5 | 1,460 | 41.32 | 700 | 40.48 |  |
|  |  |  |  |  |  |
| Birth order |  |  |  |  | 0.163 |
| 1 | 1,342 | 48.06 | 649 | 43.32 |  |
| 2 | 1,006 | 29.09 | 496 | 31.00 |  |
| 3 or more | 1,085 | 22.85 | 568 | 25.68 |  |
|  |  |  |  |  |  |
| Number of children living in the household |  |  |  |  | 0.639 |
| 1 | 1,998 | 67.43 | 920 | 64.61 |  |
| 2 | 1,066 | 27.29 | 519 | 29.80 |  |
| 3 or more | 369 | 5.28 | 161 | 5.59 |  |
|  |  |  |  |  |  |
| **Notes:** The column “n” present real sample values and the column “%” present values expanded to the Brazilian population. P value refers to chi-square test for comparisons of sample distribution of investigated variables. | | | | | |
